# Wnt/β-catenin Signaling in Normal and Cancer Stem Cells

**DOI:** 10.3390/cancers3022050

**Published:** 2011-04-19

**Authors:** Kenneth C. Valkenburg, Carrie R. Graveel, Cassandra R. Zylstra-Diegel, Zhendong Zhong, Bart O. Williams

**Affiliations:** Van Andel Research Institute, 333 Bostwick Ave. N.E., Grand Rapids, MI 49503, USA

**Keywords:** Wnt, β-catenin, stem cells, cancer stem cells, colon, mammary, prostate, therapeutics

## Abstract

The ability of Wnt ligands to initiate a signaling cascade that results in cytoplasmic stabilization of, and nuclear localization of, β-catenin underlies their ability to regulate progenitor cell differentiation. In this review, we will summarize the current knowledge of the mechanisms underlying Wnt/β-catenin signaling and how the pathway regulates normal differentiation of stem cells in the intestine, mammary gland, and prostate. We will also discuss how dysregulation of the pathway is associated with putative cancer stem cells and the potential therapeutic implications of regulating Wnt signaling.

## 1. Introduction

The “cancer stem cell” model postulates that tumors initiate from a sub-population of cancer cells that are pluripotent (cancer stem cells) and that most cells in the tumor are differentiated progeny of the cancer stem cells [1,2]. There is growing evidence that the stem cell populations in various malignancies are resistant to standard therapies that reduce the bulk of the tumor. Thus new therapies that kill these cells need to be identified. Signal transduction pathways that regulate normal stem cell activity are often deregulated during the transformation process and could provide excellent therapeutic targets. Activation of the Wnt signaling pathway is associated with expansion of several stem cell compartments and is often deregulated in human malignancies [3].

Pathways induced by Wnt ligands are highly evolutionarily conserved, with recognizable Wnt signaling pathways seen in animals as primitive as Hydra [4]. Given their strong conservation in phylogeny, it is not surprising that Wnt pathways play key roles in regulating the differentiation and pluripotency of stem cells within numerous tissues. Consistent with this, dysregulation of this pathway has been highly associated with expansion of stem and/or progenitor cell lineages in several tissues and is also highly associated with carcinogenesis [5].

**Figure 1.**
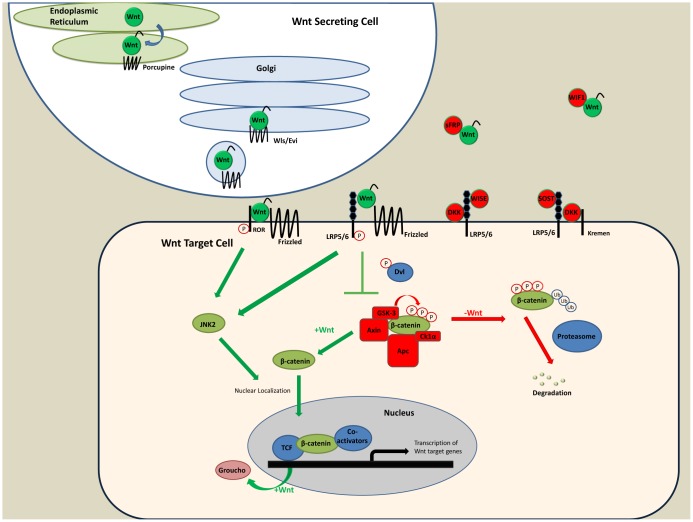
Schematic Diagram of Wnt/β-catenin Signaling Pathway.

This review will summarize our current knowledge of the mechanisms underlying Wnt signal transduction (see Figure 1) and discuss how regulation of the pathway is associated with the control of normal stem cell differentiation and how its dysregulation is associated with tumorigenesis. We will discuss the activities of the Wnt signaling pathway on the regulation of normal stem cells and tumor cells within three epithelial tissues: the intestine, breast, and prostate. This will be followed by a discussion of potential Wnt-based therapies for these and other cancers.

Wnt ligands are produced in the endoplasmic reticulum, lipid modified by the action of Porcupine, and then trafficked to the surface in a Wntless/Evi-dependent manner. Once secreted, they activated signaling in target cells via engaging a member of the Frizzled family and either LRP5 or LRP6. In the absence of a Wnt ligand, β-catenin is phosphorylated on serine and threonine residues near its N-terminus, targeting it for ubiquitin (Ub)—dependent proteosome-mediated degradation. In the presence of an upstream Wnt signal, the multi-protein complex required to target β-catenin for degradation is localized to the cell membrane via an interaction between Axin and the phosphorylated (P) C-terminus of Lrp5 or Lrp6. This inhibits the GSK3-dependent phosphorylation of β-catenin, stabilizing its levels in the cytoplasm. A concurrent signal is transmitted that results in the phosphorylation of other serine residues near the C-terminus of β-catenin, which facilitates its nuclear entry. At least in some contexts, this event is mediated by JNK2. Once in the nucleus, β-catenin interacts with a member of the LEF/TCF family of DNA binding proteins, leading to release of transcriptional repressors like Groucho proteins from target genes and the recruitment of various co-activators to the site. The net result is activation and/or repression of target gene transcription. The pathway is regulated by several other inhibitors including extracellular proteins such as Dickkopfs, secreted Frizzled-related proteins (SFRPs), sclerostin (SOST), and Wise/SOSTDC1. In addition to the pathway controlling the regulation of β-catenin stability and nuclear localization, Wnt ligands can also activate so-called “non-canonical” pathways. One example of this is the ability of Wnt5a to bind to the transmembrane receptor ROR to transduce downstream signals. *Abbreviations:* Wls/Evi (Wntless/Evenness interrupted); LRP5/6 (low density lipoprotein-related proteins 5/6); Dvl (Dishevelled); GSK-3 (glycogen synthase kinase 3); Apc (adenomatous polyposis coli); Ck1α (casein kinase 1α); JNK2 (Jun N terminal kinase 2); TCF (T cell factor - highly related to LEF1 or lymphoid enhancer factor); SOST (sclerostin).

## 2. Wnt Signaling Overview

### 2.1. Original identification of Wnt signaling

There are 19 Wnt genes found in humans. Wnts are cysteine-rich glycoproteins that share a high degree of sequence homology. The prototypic Wnt gene, *Wnt1*, was originally isolated during analysis of chromosomal insertion sites enriched in murine mammary tumors induced by mouse mammary tumor virus (MMTV) [6]. The gene was originally named int-1 (for integration site 1) and identified as a secreted protein [7]. The protein product proved difficult to work with and, during the next 14 years, a significant percentage of progress made in characterizing the protein and its downstream signaling pathways came out of work examining the genetic aspects of embryonic development in *Drosophila melanogaster*. The foundation for this work was provided by the pioneering work of Nusslein-Volhard and Wieschaus, which identified and categorized a number of genes required for embryonic development in *Drosophila* [8]. Subsequently, the Nusse laboratory reported that the *Drosophila Wingless* gene, which had been identified as being required for segment polarity in *Drosophila*, was a close homolog of the int-1 protein [9]. The fact that the *Wingless* gene had been identified as a component required for segment polarity in *Drosophila* allowed for epistasis analyses to order many components within the pathway. After subsequent work in *Drosophila* [10] and other systems (including a great deal of work in the area of *Xenopus* axis induction [11]), a consensus was reached to rename the original *Int1* gene as *Wnt1* (a combination of *Wingless* and *Int1*), with all subsequently identified homologous genes carrying the Wnt name [12].

### 2.2. Production of Wnt ligands

Several characteristics of Wnt proteins made their biochemical purification challenging. Thus, while the *Wnt1* gene was first identified in 1982, it took until 1996 to identify a putative receptor [13-15] and until 2001 to purify a Wnt protein in a biologically active state [16]. Some of this difficulty was due to how the protein is modified to facilitate secretion, including the addition of conserved palmitate [17] and palmitoleic acid [18]. Earlier genetic-based screens in *Drosophila* had shown that Wnt secretion requires the activity of the acyltransferase Porcupine [18-20]. Loss of Porcupine phenocopies the loss of Wingless in *Drosophila* embryonic development and causes accumulation of Wingless protein within the endoplasmic reticulum [21]. In addition, the secretion of Wnt ligands from cells also requires the presence of an ER-resident protein, Wntless, which binds Wnts after Porcupine-induced modification and facilitates its release from cells [22-24]. Wntless activity is also dependent on the proper function of the retromer complex which is proposed to mediate recycling of the Wntless protein back to the endoplasmic reticulum after secretion of Wnt ligands [25]. More recent work has shown the requirement for myotubularin lipid phosphatases in this process, providing another clear link to endosomal trafficking components being required for Wnt production [26].

Once secreted, Wnt ligands remain tightly associated with the extracellular matrix, with a particularly strong affinity for heparin sulfate proteoglycans [27-29]. In addition, Wnt ligands are carried on lipoprotein particles to facilitate signaling activities in the intercellular space [24,30,31].

### 2.3. Wnt receptor complexes

Wnt ligands initiate signaling pathways via engagement of several types of cognate receptors. These signaling pathways are often referred to as “canonical” and “non-canonical” pathways, although classifying Wnt ligands neatly into these categories may not be advisable [32]. The so-called “canonical” pathway, which regulates β-catenin protein levels within cells, is initiated upon engagement of a member of the Frizzled family of seven transmembrane receptor proteins in combination with either Lrp5 or Lrp6 (low density lipoprotein related proteins 5 and 6). Lrp5 and Lrp6 are members of a larger family of low density lipoprotein related receptors and most reports have focused specifically on their role in mediating Wnt signal transduction. However, roles for other members of this family, including LRP and Lrp4, in controlling Wnt signaling have also been reported [33-36]. The formation of this ligand-receptor complex results in the activation of kinases which induce phosphorylation of serine residues in the cytoplasmic tail of Lrp5 and/or Lrp6 [37]. A number of putative specific kinases have been reported to phosphorylate these residues, and the process has also been shown to be associated with activation of heterotrimeric G proteins and the cytoplasmic Dishevelled protein family [38-41]. New evidence has emerged showing that the phosphorylation and activation of Lrp6 (and potentially Lrp5) requires endocytosis and subsequent acidification of the compartment containing the endocytosed receptor. This process requires the Prorenin receptor and a vacuolar H^+^-ATPase [42-45]. Finally, binding of Wnt ligands to these receptor complexes is regulated by a number of proteins that either bind to the receptor component (such as DKKs, SOST, or Wise/SOSTDC1) or to the Wnt ligand itself (for example, SFRPs) [46-53].

### 2.4. Transduction of Wnt signals in the cytoplasm and nucleus

The phosphorylation of the cytoplasmic tail of Lrp6 leads to the recruitment of the scaffolding protein Axin to the receptor complex [37,54]. This recruitment is facilitated by the phosphorylation of multiple copies of this phosphorylated proline-rich serine motif in each Lrp6 molecule and via potential clustering of multiple Lrp6 receptors upon activation [55,56]. Axin is a component of a multi-protein complex that, in the absence of an upstream signal, is responsible for inducing the degradation of the β-catenin protein. Other components of this complex include the colon cancer tumor suppressor protein Apc, and the serine/threonine protein kinase glycogen synthase kinase 3 (GSK3). In un-stimulated cells, Serine-45 in the β-catenin protein is constitutively phosphorylated within the cytoplasm, which creates consensus sites for GSK3 to mediate further phosphorylation of β-catenin [57]. This hyperphosphorylated form of β-catenin is then targeted for ubiquitin-dependent proteolysis [58]. In addition to the Wnt-induced pathway that inhibits degradation of β-catenin via inhibition of GSK3 activity, a parallel pathway is induced by the receptor complex which facilitates nuclear entry of β-catenin from the cytoplasm. One report indicates that a PI3K-dependent pathway acting via Rac1 and JNK2 is necessary for nuclear entry of β-catenin [59]. Another report found that activation of the Ras signaling pathway can also facilitate this process [60].

Once β-catenin enters the nucleus, it interacts with members of the LEF/TCF family of DNA binding proteins to bind to specific promoter targets [61]. β-catenin/LEF/TCF complexes have been shown to interact with a variety of nuclear factors to control specific transcriptional targets. Examples of such proteins include p300, CBP, Hrpt2, Foxo, Bcl9-2, reptin, pontin, Grouchos, Prmt2, and CtBP [62]. One result of such interactions is the reorganization of chromatin near the transcriptional initiation site of target genes. For a thorough review of this process, please see Mosimann *et al.* [62].

### 2.5. β-catenin-independent signaling pathways induced by Wnt ligands

In addition to regulating the cytoplasmic stability of β-catenin, Wnt-induced inhibition of GSK3 can also directly regulate the activity of the mTOR pathway [63]. In the absence of an upstream Wnt signal, GSK3 phosphorylates TSC2, which normally acts to inhibit cell growth by activating the GTPase activity of Rheb, causing inhibition of mTOR. Inhibition of TSC2 results in Rheb activation and downstream activity of mTOR. The relative consequences of mTOR to Wnt-associated phenotypes will be of continued interest. For example, at least two recent reports have found that mTOR activation associated with Wnt signaling plays a dominant role in phenotypes seen in mouse models [64,65].

Recent work has also greatly increased our knowledge regarding pathways downstream of Wnt ligands that are independent of GSK3 regulation. As stated previously, these diverse pathways have historically been referred to as “non-canonical” Wnt signaling. These include pathways that control the activation of protein kinase C (PKC), protein kinase A, the small GTPase RhoA, and several others [66]. In some contexts, these pathways not only activate cellular responses independent of the Wnt/GSK3 pathway, but also inhibit signaling through the β-catenin protein. One particularly interesting example is the recent demonstration that the Wnt5a protein can simultaneously bind to Frizzled proteins and the receptor tyrosine kinase ROR2, resulting in the phosphorylation of the cytoplasmic tail of ROR2 (analogous to the Wnt-induced phosphorylation of Lrp6) [67]. It is possible that the competition for Frizzled proteins between Wnt/ROR2 and Wnt/LRP6 could cause inhibition of β-catenin activation via Wnt ligands. In addition, Wnt5a signaling via PKCα leads to phosphorylation of serine 35 in the orphan nuclear receptor RORα. Phosphorylated RORα then binds directly to β-catenin to mediate repression of Wnt/β-catenin target genes [68]. In addition, the demonstration that another Wnt receptor, Ryk, undergoes a cleavage process to liberate its cytoplasmic domain to eventually enter the nucleus is another example of diverse responses downstream of Wnt ligands [69].

## 3. Wnt Signaling in the Intestine

The intestinal epithelium is regenerated throughout adult life with robust proliferation. The mammalian intestine is lined by a monolayer of epithelial cells (or mucosa), which contains a crypt compartment at the base maintained by multipotent stem cells and a villus compartment facing the lumen. In the colon, there is a flat surface epithelium instead of a villus compartment. The crypt contains a group of undifferentiated and actively dividing cells with stem cell properties that will eventually invaginate into the mucosa to form crypts and villi [70,71]. Epithelial cells located at the +4 position (4 cells up from the crypt bottom) can be labeled with ^3^H-TdR DNA labels throughout long periods of time, which means that +4 cells are either in a prolonged quiescent state or are in a slow-cycling state. In addition, the +4 cells are extremely sensitive to radiation [72,73]. Therefore, intestinal stem cells were proposed to localize at the +4 positions and give rise to all other cell types in the epithelium.

### 3.1. Wnt signaling in intestinal stem cells

It is well accepted that Wnt/β-catenin signaling plays a key role in the homeostasis and maintenance of intestinal stem cells [70,71]. There is a Wnt signaling gradient from the crypt base to the villus [71]. When epithelial cells are far away from the Wnt source at the crypt base, they lose the proliferative capacity and differentiate. Depletion of Wnt downstream genes [74,75] or co-receptors Lrp5/6 (unpublished data, B. O. Williams) in the intestine blocks stem cell activity and the proliferation of the intestinal epithelium. The regions between the villi, which normally contain highly proliferative cells, were found to be entirely composed of non-cycling differentiated cells. Ectopic expression of Wnt inhibitors (such as DKK1) by means of an adenovirus infection or transgenic technology in a mouse resulted in the loss of crypt cells and decreased villus size and number [76]. In the adenovirus-mediated DKK1 overexpression model, decreased DKK1 expression at later time points rescued the phenotype [77]. Numerous studies suggest that the tremendous self-renewal through active proliferation in crypt stem cell compartments is dependent on Wnt signaling. Most of the putative intestinal stem cell markers indentified so far are Wnt target genes, such as Msi1, CD44 and Lrg5 [78-80]. Lgr5-positive intestinal stem cells reside between Paneth cells (intestinal epithelial cells) at the small intestinal crypt base and divide daily [81]. A single Lgr5-positive stem cell can build crypt-villus structures *in vitro* [82]. Lgr5 marks colon stem cells as well, whereas only 10% of Lgr5-positive cells were observed at the +4 position [79]. A recent study showed that Lgr5-positive stem cells divide symmetrically and compete with the stem cell population for the “stem cell niche,” rather than asymmetrically at the single stem cell level to maintain crypt homeostasis [81]. Therefore, Wnt signaling is a key role player in maintaining intestinal stem cell population.

### 3.2. Wnt signaling in intestinal cancer stem cells

The majority of colorectal cancer (CRC) is caused by mutations in key components of the Wnt signaling pathway [83-85]. The adenomatous polyposis coli (*APC*) gene is a well-known tumor suppressor that plays a central role in the Wnt signaling pathway by targeting β-catenin for degradation. Germline loss-of-function mutations in the *APC* gene were originally identified to be associated with familial adenomatous polyposis (FAP), about 1% of which progress to CRC [86-88]. Furthermore, 85% of cases of sporadic intestinal neoplasia have mutations in APC, while activating mutations in β-catenin were found in approximately 50% of CRC tumors lacking APC mutations [89]. Although the dysregulation of β-catenin caused by APC loss may lead to colon tumor initiation, several studies have failed to detect nuclear accumulation of β-catenin (as an indicator of Wnt activation) upon APC mutation in early human colon adenomas. Recently, it was shown that β-catenin stabilization and C-terminal binding protein 1 (CtBP1) following APC inactivation contribute to adenoma initiation as the first step, and that KRAS activation and β-catenin nuclear localization act synergistically to promote adenoma progression to carcinoma [60].

Several mouse models of FAP and intestinal carcinoma have been generated by inducing mutations at different amino acid positions in APC [90-93]. The renowned APC^Min/+^ mice are so-called because the heterozygous APC mutation induces multiple intestinal neoplasias, while homozygous APC^Min/Min^ mice die at gastrulation. The APC^Min/+^ mouse was originally identified in chemical mutagenesis screens and proved to carry a premature stop/truncation mutation in the *Apc* gene [87,93]. The APC^Min/+^ mice raised on a high fat diet developed adenomas throughout the intestinal tract and mostly died by 120 days of age. The presence of multiple lineages in the adenomas of APC^Min/+^ mice suggests that tumorigenesis may be initiated in a multipotent stem cell [94].

Until recently, it was not clear which cell type sustained the cancer-initiating mutation, but due to the well-determined stem cell markers of the intestine, significant progress has been made in identifying the origin of intestinal adenomas induced by activated Wnt signaling. Critical work by Barker *et al.* showed that Apc inactivation in Lgr5-positive stem cells at the crypt bottom could lead to transformation within days [95]. In contrast, APC inactivation in progenitors or differentiated cells did not cause tumor formation even after 30 weeks [95]. These studies indicate that the cellular origin of CRC initiation might be within normal stem cells of the intestine, rather than progenitors or differentiated cells. Another study demonstrated that severe polyposis in Apc loss-of-function mutant (Apc1322T) mice was associated with increased expression of the stem cell marker Lgr5 and other stem cell markers (Musashi1, Bmi1, and the Wnt target CD44) [96]. Furthermore, the Wnt target gene CD44 has been identified as a marker for colorectal cancer stem cells, and deletion of CD44 in APC^Min/+^ mice attenuates intestinal tumorigenesis [97,98]. Overall, these studies support the cancer stem cell model in intestinal tumorigenesis and that Wnt signaling plays a key role in this progression.

Many studies have shown that inhibition of Wnt signaling can reverse tumorigenic properties of CRC cells. The inhibition of the β-catenin interaction with its transcription factor TCF in colon cancer cells caused an arrest of the cell cycle in the G1 phase [99,100]. These studies suggested that this cell cycle phenotype was due to the regulation of genes such as cyclin D1 and p21/CIP1. The promoter of secreted Frizzled-related protein (*SFRP*) is hypermethylated often in CRC, and this epigenetic silencing contributes to overactive Wnt signaling, as evidenced when reintroduction of SFRP to colon cancer cells reversed Wnt signaling [101]. This study suggests the therapeutic possibility of activating *SFRP* by silencing the gene that induces its promoter methylation. Another example is the inhibition of DACT3 (a member of the Dpr/Frodo family) methylation. DACT3 is a negative regulator of Wnt signaling that has its expression suppressed in CRC via histone methylation and deacetylation [102]. When DACT3 expression was restored by the inhibition of these histone modifications, it resulted in reduction of Wnt signaling and induction of CRC cell apoptosis. Many other Wnt repression strategies exist as therapeutic options for CRC, and we will discuss these in a later section.

## 4. Wnt Signaling in the Mammary Gland

The mammary gland is a complex organ that undergoes the majority of development postnatally and extensive tissue remodeling during reproductive estrous cycles. These unique features, in addition to the ability to perform epithelial reconstitution assays, have allowed a more thorough analysis of stem cell differentiation within the mammary gland. Recent studies have identified several mammary stem cell (MaSC) surface markers that have laid the groundwork for understanding the hierarchical organization of the mammary epithelium. In mice MaSCs are highly enriched in the CD49f^hi^CD29^hi^CD24^+^Sca1^-^ subpopulation [103-105], whereas human cells with features of MaSCs have been identified in the CD49f^hi^EpCAM^-^ subpopulation [106,107]. In addition, there are several established mouse mammary models that have revealed the importance of several signaling pathways in both normal mammary development and mammary tumorigenesis. In this section we will discuss the critical role that Wnt signaling plays in both normal mammary stem cells and cancer stem cells (CSCs).

### 4.1. Wnt signaling in mammary stem cells

Several studies in mice have revealed the integral role of Wnt signaling in the development of the normal mammary gland. Numerous components of the Wnt signaling cascade are expressed during embryonic mammary morphogenesis, including Wnt ligands (*i.e.*, Wnt1, Wnt2, Wnt3, Wnt3a, Wnt5a, Wnt5b, Wnt6, Wnt7b, Wnt10a, Wnt10b, Wnt11), receptors (*i.e.*, Fzd1-9, Lrp5, Lrp6), and downstream DNA-binding proteins (*i.e.*, Tcf1, 3, and 4 and Lef1) [108]. Mammary development initiates on embryonic day 10.5 (E10.5) with the formation of two mammary lines which give rise to five mammary placodes. The placodes grow through the dermis and colonize the fat pad [109]. Embryos overexpressing the Wnt antagonist Dkk1, as well as animals deficient for Lrp6 or Lef-1, fail to form mammary placodes [110-112]. These findings validate the importance of Wnt/β-catenin in mediating the activity of mammary stem cells.

At the time of birth, the rudimentary gland consists of ducts containing two differentiated cell types, an inner layer of luminal epithelial cells, which secrete milk, and myoepithelial cells, which are located on the basal surface of the luminal cells adjacent to the fat pad. Most of the development of the mammary gland takes place postnatally and depends on cues from the hormonal and local microenvironment. In juvenile animals, the tips of growing ducts have a club-shaped structure known as the terminal end bud, which is enriched in stem cells. The hormonal environment stimulates active proliferation of the terminal end bud and induces invasion of the surrounding adipose tissue to form complex branching structures. Werb *et al.* have shown that Wnt2, Wnt5a and Wnt7b expression is enriched in the terminal end bud microenvironment [113]. Knockout mouse models of Lrp5 and Lrp6 display fewer terminal end buds and delayed ductal expansion [110,114]. Conversely, animals deficient for Wnt5a in the mammary gland have larger terminal end buds and increased ductal elongation and lateral branching [115]. Given that Wnt5a is considered a non-canonical Wnt, its role may be to negatively regulate the Wnt/β-catenin pathway during mammary development. On the other hand, constitutive expression of the canonical Wnt4 leads to more highly branched ducts in virgin females, similar to what occurs during early pregnancy [116]. These studies demonstrate that Wnt signaling is crucial for the normal mammary gland development.

During estrous cycles, pregnancy, and lactation, there is an expansion of the mammary epithelium, and during involution, extensive remodeling reduces the gland to a pre-pregnancy state. The hormonal and local factors that orchestrate these changes during development are still marginally understood. In the luminal epithelium of the mammary duct, cells are either positive or negative for both the estrogen receptor α (ERα) and progesterone receptor (PR), while the basal, myoepithelial cells express neither receptor [117]. Despite lacking both ERα and PR, MaSCs are highly responsive to steroid hormone signaling [118,119]. During pregnancy there is a marked increase in the number of MaSCs, and hormone deprivation reduces the activity of these cells [118]. Recent studies demonstrate that MaSCs are hormonally influenced by progesterone. Progesterone induces the secretion of ligands, such as Wnt4 and receptor activator of nuclear factor kappa-B ligand (RANKL), which act as paracrine effectors on MaSCs [118,119]. Further evidence for the direct effect of Wnt ligands on MaSCs comes from the Nusse laboratory, where the addition of Wnt3a protein to MaSCs stimulates clonal MaSC expansion for many generations [120]. These studies and others demonstrate the significant role that Wnt signaling plays in both mammary gland development and hormonally induced mammary remodeling. Further investigation is needed to fully understand how both canonical and non-canonical Wnt signaling impacts MaSCs.

### 4.2. Wnt signaling in breast cancer and breast cancer stem cells

Studies in both mouse models and human breast cancers have revealed that Wnt signaling is critical to mammary tumorigenesis. Wnt signaling was first implicated in mammary tumors when the mouse mammary tumor virus (MMTV) was found to integrate into the *Int-1* (*Wnt1*) locus, and overexpression of Wnt1 induced mammary tumorigenesis [9,121]. Mouse models have revealed that aberrant Wnt signaling induces mammary carcinomas with diverse pathologic phenotypes [122]. In addition, Teissedre *et al.* have demonstrated that distinct Wnt signaling components affect distinctive mammary cell populations [123]. MMTV-ΔN89β-catenin activated signaling within luminal progenitors whereas MMTV-Wnt1 signaling was observed in basal cells. In 2003, Li *et al.* demonstrated that aberrant Wnt signaling alters MaSC populations during tumorigenesis [6]. An expanded MaSC population was observed in MMTV-Wnt1 mammary tumors, yet this population was not observed in mammary tumors isolated from MMTV-Neu, MMTV-H-Ras, or MMTV-PyMT mice. This expanded MaSC population was also found in other mouse models of downstream Wnt targets such as MMTV-β-catenin and MMTV-c-myc. Further studies by the Visvader laboratory demonstrated that constitutive Wnt signaling results in an expanded progenitor cell population during preneoplastic growth [124]. Using the luminal marker CD61/β3-integrin as a CSC marker, CSC populations were identified within MMTV-Wnt-1 tumors, yet not in MMTV-Neu tumors. These studies indicate that Wnt signaling may affect various mammary epithelial cells and induce an expansion of stem-like cells during tumor progression.

In spite of the strong evidence for Wnt signaling in mouse mammary tumor models, there are conflicting reports on the importance of Wnt activation in human breast cancer. Numerous reports have identified dysregulation of the Wnt pathway in breast cancer [125-130]; however, others have failed to find an association with metastasis or clinical outcome [131-133]. More recent studies have evaluated Wnt signaling in the context of the recently identified breast cancer molecular subtypes (luminal, Her2, basal, normal-like). This revealed that aberrant β-catenin expression was associated with basal and triple-negative breast cancers and poor clinical outcome [134]. Furthermore, Lindvall *et al.* have demonstrated that overexpression of Lrp5 also correlates with basal breast cancers [110]. Down-regulation of the secreted Frizzled-related proteins (SFRPs), which are Wnt inhibitors, has been observed in breast cancers [135-137]. In many cancers, mutations in the Wnt pathway are commonly found, but they are less common in breast cancer. Mutations within APC have been identified in 18% of breast cancers, however mutations have not been identified in *CTNNB1* (the gene encoding β-catenin) [138,139]. Therefore, aberrant Wnt signaling occurs in specific breast cancer subtypes, such as basal-like, and is not directed by mutational activation.

Studies in breast cancer cell lines have shown that stem cell populations are more resistant to radiation treatment and that Wnt/β-catenin signaling mediates resistance [140-142]. Recent work by the Rosen lab evaluated radiation resistance in CSCs isolated from p53-null mouse mammary tumors [143]. These CSCs populations had altered DNA repair in response to radiation and increased AKT (a serine-threonine protein kinase) and β-catenin activation. By using the inhibitor perifosine, they were able to block AKT and β-catenin activation and sensitize the cells to radiation. This study and numerous others underscore the importance of gaining a clear understanding of Wnt signaling in breast cancer for the development of effective therapeutics.

## 5. Wnt Signaling in the Prostate Gland

The function of the adult human prostate gland is to secrete proteolytic enzymes into the semen to facilitate conception. The prostate (which surrounds the urethra at the base of the urinary bladder) develops out of the embryonic urogenital sinus (UGS), a derivative of the endoderm layer. Testosterone production begins in the human fetus about 8 weeks into gestation, and at 10-11 weeks, solid epithelial buds begin to extend and branch into a complex series of ducts that form the adult prostate and open into the urethra [144]. The anatomical development of the prostate has been much debated over the past century, and the accepted model for prostate anatomy is that it develops as a zonal structure (proposed by McNeal), rather than a lobular structure [144]. Prostatic ducts consist of three cell types: secretory luminal epithelial, basal epithelial, and rare neuroendocrine cells. Prostate epithelial cells may differentiate from basal-like cells, luminal-like cells, or both [145-148]. Neuroendocrine cells can be found at the urogenital bud outgrowths at 11 weeks, but epithelial differentiation does not take place until weeks 13-16 of human embryonic development [149]. There are three major stages of epithelial differentiation: embryonic development in which embryonic-like cells express both basal and luminal markers; a pre-puberty stage that consists of quiescent immature epithelial layers; and sexual maturity, when the prostate has distinct, fully differentiated epithelial layers [150].

Full development of the prostate depends on the presence of androgens, which are secreted by the testes during puberty. Androgens bind to and induce the homodimerization and nuclear translocation of the androgen receptor (AR), thereby causing expression of target genes such as prostate-specific antigen (*PSA*), transmembrane protease serine 2 (*TMPRSS2*), and others [151-153]. AR expression in the mesenchyme surrounding prostate tissue is sufficient to induce mature prostate formation [154]. Androgen deprivation via physical or chemical castration results in involution of the prostate and apoptosis of approximately 90% of prostate luminal cells [155]. Therefore, androgen deprivation therapy is one of the standards of prostate cancer therapy, although many patients become resistant to this therapeutic strategy.

### 5.1. Wnt signaling in prostate development and stem cells

The fate of prostate cells is highly regulated by various molecular signaling pathways, such as Notch, AR, Nkx3.1, Hedgehog, and Wnt [156]. Recently, the Wnt pathway has been implicated in prostate differentiation. Wnts were first found to be differentially expressed during embryonic prostate development by serial analysis of gene expression (SAGE) libraries [157]. Wnt4 in particular was highly expressed during embryonic development and significantly less so in the adult prostate. In addition, the Wnt antagonist secreted Frizzled-related protein 2 (SFRP2) was highly expressed early in development and was down-regulated at later time points. These studies indicate that Wnt signaling is temporally regulated during prostate development and induces cell fate changes of prostate progenitors. In another study, rat prostate organ cultures were treated with Wnt3a or the Wnt inhibitor Dikkopf1 (DKK1) [158]. The resulting cellular morphology of the cultures was different, in that Wnt3a-treated cultures had enlarged end buds, and the DKK1-treated cultures had smaller and fewer ducts. This indicates that both enhancement and reduction of Wnt signaling can adversely affect branching morphogenesis. Wnt3a-treated cultures were enriched for basal cells (which have been shown to be enriched for putative prostate stem cells), whereas DKK1-treated cultures showed lower staining for basal cell markers. Finally, Wnt3a RNA was highly expressed early in development and decreased in an incremental fashion over time. Non-canonical Wnt5a transcripts were also found in embryonic prostatic buds [159]. Wnt5a-null mice displayed multiple developmental defects of the prostate and urogenital sinus. Further studies using *ex vivo* cultures and Wnt5a-null mice indicated that Wnt5a represses luminal epithelial differentiation [160].

### 5.2. Wnt signaling in prostate cancer and cancer stem cells

Wnt pathway members have been widely studied in prostate cancer in the past decade [161-163]. It has been hypothesized that prostate cancer cells adopt embryonic signaling pathways (such as Wnt) that are generally silent in differentiated cells [164]. Wnt ligands are up-regulated in prostate cancer, and their expression often correlates with aggressiveness and metastasis. Keller *et al.* determined that 15 of the 19 Wnts are expressed in four prostate cancer cell lines [165]. Elevated expression levels of Wnt1, Wnt5a, Wnt7b, and Wnt11 have also been correlated to prostate cancer aggressiveness [166-169]. In addition, DKK1 expression increases during prostate cancer initiation but decreases during metastasis [170]. The correlation of Wnt activation and skeletal metastasis may be important for therapy; there is currently no cure for metastatic prostate cancer.

Other Wnt pathway members are dysregulated in prostate cancer. Frizzled-4 (FZD4, a Wnt receptor) is co-expressed in human prostate tumor samples with the ETS-related gene (*ERG*) [171]. Further experimentation has shown that FZD4 overexpression decreases E-cadherin expression in ERG-positive prostate cancer and leads to an epithelial-to-mesenchymal transition (EMT), which is a crucial step in metastasis initiation. Other studies have shown that Wnt inhibitory factor-1 (WIF1) is down-regulated in prostate cancer [172], and induced overexpression of WIF1 reverses EMT in prostate cancer cell lines and decreases their invasive capacity *in vitro* and *in vivo* [173]. Also, when prostate cancer cell lines were transfected with WIF1, they were more sensitive to chemotherapy and had reduced phosphorylation of Akt (a key effector of PI3K signaling which is frequently phosphorylated in prostate cancer) [174]. Furthermore, when cells deficient in DKK1 were injected into the tibiae of severe combined immunodeficient (SCID) mice, the up-regulated Wnt signaling induced an osteoblastic phenotype, suggesting that Wnts may be important in osteoblastic skeletal metastases from prostate tumors [165].

Conditional mouse models have shown that aberrant Wnt signaling in the prostate is tumorigenic. The prostate epithelial-specific gene for Probasin (*PB*) has been widely used to control Cre recombinase expression and subsequently activate or inactivate genes flanked by loxP regions [175]. In many cases of prostate cancer, adenomatous polyposis coli (*APC*) is mutated and hypermethylated to a silent form, and β-catenin is also frequently mutated to an active form [176-178]. A mouse model in which the *Apc* gene was inactivated (referred to as *PB^Cre/^*^+^*Apc^flox/flox^*) resulted in prostatic hyperplasia and adenocarcinoma [179]. Another mouse model incorporated activated β-catenin in the prostate (known as *PB^Cre/^*^+^*Catnb^lox(ex3)^*), and adenocarcinoma also developed in these mice [180,181]. More work remains to be done to determine the full range of involvement of Wnt signaling in prostate cancer, but it has been suggested that Wnt inhibition represents a valuable chemoprevention approach for the disease [182].

Studies using specific cell markers have identified prostate cancer stem cells (PCSCs), which are prostate cancer cells with stem-like properties [148,183-186]. It would be of great interest to determine the level of Wnt activation in these cell populations. All the evidence that Wnt signaling can induce prostate cancer initiation, EMT, and metastasis suggests that Wnts may play a role in PCSCs. Furthermore, Wnt3a treatment increased the self-renewal of putative PCSCs independent of androgen signaling, and Wnt11 expression was shown to be inversely correlated to AR expression in prostate cancer cell lines and primary cultures [187,188]. Wnt signaling has also been suggested to crosstalk with AR and induce its translocation to the nucleus, thereby enhancing the expression of AR target genes independent of androgen ligands [189]. This suggests that aberrant activation of Wnt signaling in prostate cells may cause them to become PCSCs having the ability to stimulate AR target genes regardless of androgen status. Indeed, nuclear localization of β-catenin has been positively linked to androgen deprivation therapy failure in patients [190]. The fact that Wnt signaling may augment AR signaling has implications for therapy due to the frequent recurrence of prostate cancer after androgen deprivation therapy. If Wnt inhibition can be confined in a targeted manner to PCSCs, it may be possible to treat castration-resistant prostate cancer, which is currently incurable. Further studies are necessary to determine how Wnt signaling regulates PCSCs in the various stages of tumor progression.

## 6. Potential for Wnt Antagonists as Cancer Therapeutics

As discussed thus far, Wnt signaling is active in intestinal, mammary, and prostate cancers, which consist primarily of epithelial tissue. Wnt has also been implicated in skin cancer, lung cancer, bladder cancer, and leukemia, and it may be oncogenic in other cancers [3,191-193]. Therefore, Wnt inhibition could represent a valuable strategy for cancer therapy [193]. We have already discussed several specific examples of the benefits of Wnt inhibition in some cancers. The wealth of evidence that cancer cells with stem-like characteristics (CSCs) may be the cells of origin for many cancers indicates that targeted Wnt inhibition in those cells would be most beneficial [3,191,194]. There are several ways that the Wnt pathway can be abnormally activated in cancer, due the large number of proteins involved in the pathway [192]. For this reason, there is great potential for the development of a wide array of Wnt antagonists. Several classes of drugs that target the Wnt pathway are currently on the market or are under development. These drug categories include non-steroidal anti-inflammatory drugs (NSAIDs), vitamin D derivatives, antibody-based treatments, and small molecule inhibitors.

### 6.1. NSAIDs

NSAIDs reportedly have some anti-cancer effects, particularly in colon cancer, and it is widely believed that the mechanism is related to the inhibition of cyclooxygenase (COX) activity. However, this may only be part of the answer, as COX and Wnt signaling appear to be linked [195]. It was first suggested that NSAIDs inhibited Wnt signaling when sulindac was used to treat familial adenomatous polyposis (FAP, a disease caused by inactivating mutations in the Wnt inhibitor APC) to reverse polyp growth [196]. This observation was validated when sulindac sulfide reduced nuclear β-catenin levels in FAP polyps [197]. Aspirin has also been implicated in Wnt inhibition via interference with the interaction of β-catenin with the transcription factor T cell factor (TCF) [198]. While NSAIDs may have some benefit for cancer treatment, they have not been indisputably proven as anti-cancer drugs.

### 6.2. Vitamin D

Vitamin D (or at least its analog EB1089) may protect against some cancers. Colon cancer xenograft studies, in which nude mice were treated with EB1089, have shown that tumors decreased in volume due to an increase of apoptosis [199-201]. The molecular mechanism by which vitamin D accomplishes this feat is unknown, but it has been hypothesized that it is through inhibition of β-catenin [202]. The absence of β-catenin abrogates the ability of vitamin D to activate its receptor (vitamin D receptor, or VDR), suggesting that β-catenin and VDR directly interact [203]. These studies imply that increasing the amount of vitamin D in one's diet might prevent certain cancers, with the caveat that some cancers have lost expression of VDR, thereby limiting its utility [193]. Other chemopreventive agents, such as lycopene or curcumin, may also have Wnt inhibitory effects, although their molecular mechanisms are not yet fully elucidated [204].

### 6.3. Antibody-based treatments

Endogenous Wnt inhibitors such as sclerostin, DKK1, Wise, and Mesd bind to and inactivate the Wnt co-receptors Lrp5 and Lrp6 [48,50,51,205,206]. To imitate this natural Wnt inhibition, Lrp6 antibodies have been synthesized by two different labs in the past year [207,208]. Two classes of Lrp6 antibodies were discovered by Cong *et al.*, one that inhibited Wnt1-Lrp6 interactions and one that inhibited Wnt3a-Lrp6 interactions [207]. These antibodies were able to block tumor growth in Wnt1- and Wnt3a-driven xenografts. The downside of these antibodies is that they may sensitize cells to other up-regulated Wnts. Therefore, these particular antibodies may only be useful in cancers that are specifically driven by Wnt1 or Wnt3a. Costa *et al.* also discovered two novel Lrp6 antibodies that inhibited Wnt3- and Wnt3a-Lrp6 interactions [208]. When Wnt-driven tumors were treated with these antibodies, there were mixed results: one antibody induced tumor regression, while the other showed no effect on tumor volume. This may be because the tumors are not being driven specifically by Wnt3 isoforms or because the antibodies also have a potentiating effect on some Wnt isoforms. Further investigation is required to determine the full range of effects of these antibodies.

Jablons *et al.* has done a great deal of work with Wnt1 and Wnt2 antibodies in various cancers [209-213]. Wnt1 monoclonal antibodies induce apoptosis of cancer cells *in vitro* (lung, breast, and mesothelioma cell lines) and *in vivo* (injection of lung cancer cells into nude mice, treated by Wnt1 antibodies) [209,213]. This phenotype was similar to that seen with RNAi knockdown of Wnt1, indicating that Wnt1 is important for cancer cell survival. A monoclonal antibody against Wnt2 was shown to induce apoptosis *in vitro* in lung cancer, mesothelioma, and malignant melanoma [210-212]. Also, Wnt2 antibody treatment in nude mice with melanoma tumors stopped tumor growth [211]. Other Wnt pathway members, such as the Frizzled receptors, are options for future antibody development [214].

### 6.4. Small molecule inhibitors

Several Wnt pathway members have been targeted for inhibition via small molecule inhibitors. β-catenin interacts with the TCF transcription factor to induce expression of target genes (particularly *c-myc* and *p21/CIP1*), and this interaction is one such target [215]. While these inhibitors have been shown to abrogate Wnt signaling, there may also be off-target effects because β-catenin is also important in cellular adhesion, and TCF interacts with other signaling pathways [215]. Therefore, more work needs to be done with this type of inhibitor to enhance the specificity to nuclear β-catenin. Another inhibitor (ICG-001) has been found to selectively antagonize interactions between β-catenin and the cyclic AMP response element-binding protein (CBP), which is a transcriptional co-activator essential for β-catenin-mediated transcription [216]. This particular inhibitor may be used in clinical trials for leukemia in the near future [217]. A third molecule (NSC668036) mimics the endogenous Wnt inhibitor Dapper1 by inhibiting the interaction of Disheveled with Frizzled [218,219]. Axin is a member of the destruction complex that induces ubiquitin-mediated degradation of β-catenin, and the inhibitor XAV939 stabilizes Axin by tankyrase inhibition, thereby reducing Wnt signaling [220]. Casein kinase 1α (CK1α) is similar to Axin in that it plays a role in β-catenin degradation, and pyrvinium selectively activates CK1α [221]. Downstream targets of Wnt that have been implicated in carcinogenesis, such as c-Myc or cyclin D1, can also be silenced with small molecule inhibitors [193]. RNA interference (RNAi) screens are currently being employed to discover more novel regulators of the Wnt pathway [222]. None of these Wnt inhibitors have yet been used in the clinic due to dosage and toxicity issues, but there is certainly hope of future progress as biotechnology improves and off-target effects are reduced [193,217].

It is important to note that disruption of the Wnt pathway may result in a variety of phenotypes in assorted tissues throughout the body, so care must be taken in the growing field of anti-Wnt therapeutics. For example, in lung cancer, one study showed that antibodies against the Wnt inhibitor DKK1 inhibited cell growth and invasion [223]. Osteoblast-mediated bone formation is enhanced by nuclear β-catenin [224], and a recent study shows that neutralization of DKK1 protects from bone loss [225]. However, Wnt antagonists show promise in cancer therapy. The goal is to develop safe and effective Wnt inhibitors that target cancer cells or CSCs and do not affect normal cells or stem cells. Therefore, the challenge will be to continue to ascertain the different tissue- and cell-specific signaling that can take place via the 19 Wnt ligands and the numerous downstream pathway members.

## 7. Summary

The key role of Wnt/β-catenin signaling in regulating the differentiation of stem cell populations, coupled with the fact that dysregulation of this pathway is associated with numerous tumor types, makes it an attractive target for anti-cancer therapeutics. While this review has focused on these concepts in the context of three of the most common tumor types which are epithelial in origin, there is also substantial evidence demonstrating key roles for this pathway in hematopoietic malignancies and in other solid tumors (for example, see [226] and [227]). Given that several pharmaceutical and biotechnology companies have substantial programs designed to target this pathway to treat various human diseases [228], it is likely that such agents will see expanded testing in clinical trials for several human diseases.
